# Financial Literacy, Financial Education, and Cancer Screening Behavior: Evidence from Japan

**DOI:** 10.3390/ijerph19084457

**Published:** 2022-04-07

**Authors:** Trinh Xuan Thi Nguyen, Sumeet Lal, Sulemana Abdul-Salam, Mostafa Saidur Rahim Khan, Yoshihiko Kadoya

**Affiliations:** School of Economics, Hiroshima University, 1-2-1 Kagamiyama, Higashihiroshima 7398525, Japan; xuantrinh93@gmail.com (T.X.T.N.); sumeetshivamlal@yahoo.com (S.L.); salamyoo@yahoo.com (S.A.-S.); ykadoya@hiroshima-u.ac.jp (Y.K.)

**Keywords:** cancer screening, financial literacy, financial education, Japan

## Abstract

Although Japan has a well-established cancer screening program and has implemented several initiatives to increase screening rates, levels of cancer screening can be further improved. Based on a rational decision-making framework, this study examines the role of financial literacy and financial education, which measure peoples’ knowledge about investment and savings, respectively, in improving cancer screening rates in Japan. The main data were extracted from Osaka University’s Preference Parameters Study for 2011. The dependent variable was the number of cancer screenings while the two main independent variables were financial literacy and financial education. Ordered probit regression models were run to test the association between financial literacy, financial education, and the number of cancer screenings. The results showed a positive relationship between financial education and cancer screening behavior in Japan, while no significant association was observed between financial literacy and screening behavior. Furthermore, according to findings stratified by three age groups, the positive association between financial education and cancer screening behavior was particularly evident in 50- to 59-year-olds, while the effects of other demographic, socioeconomic, and risky health behavior variables were not consistent. It is imperative that implementation of more financial education programs is an effective intervention to encourage cancer screening behavior in Japanese populations.

## 1. Introduction

Although Japan has a well-established cancer screening program [1,2] with several initiatives to increase screening rates [3,4], the unexpected flattening trend in cancer screenings shows room for improvement [5]. The mortality rates of the five cancers regulated under the national cancer screening guideline have shown little improvement [6]. Therefore, ways to increase people’s attendance rates in cancer screening examinations and identifying factors associated with this behavior are worth careful consideration. Since the monetary costs of basic cancer screening are widely covered in Japan [7], participation in preventive cancer screening seems to depend more on people’s rational health behavior. In this study, we use financial literacy and financial education as proxies to observe cancer screening behavior from the rational decision-making perspective. We use financial literacy to measure financial knowledge from the viewpoint of investment while financial education concentrates on savings behavior.

We consider that the lack of participation in preventive cancer screening can be explained from the perspective of an irrational choice framework and offer a solution based on rational decision-making abilities using previous studies that associated financial literacy and financial education with rational health behavior [8,9,10]. Following Grossman’s health capital model [11,12], preventive cancer screening could be conceived as an investment in health capital, as one in every six deaths is caused by cancer [13]. Therefore, rational people are likely to participate in cancer screening to reduce the risk of potential illness and to remain productive. However, previous studies have found that cognitive limitations might interfere with people’s intention to be screened for cancer [14,15], and the rationality of cancer examination behavior is partly affected by emotions and feelings [16,17]. Being able to understand the value of health capital, financially literate people are likely to overcome cognitive limitations and become motivated for preventive cancer screening. Previous studies also provide evidence that financial literacy aids people in making rational choices about savings, finance, and investment [18,19], planning for retirement [20,21,22,23], and health behavior [8,9,10,24]. Furthermore, financial literacy is associated with an improved cognitive performance and aids time-consistent decisions about current and future outcomes [25].

Our study examines whether financial literacy and financial education are associated with preventive cancer screening in Japan. We hypothesize that financial literacy and financial education, which enable people to make rational decisions, increase the likelihood of participation in preventive cancer screening. As many cancers are asymptomatic in their early stages and the symptoms may not be visible until the end stage [26], the decision to not be screened for cancer early and regularly would be regarded as an irrational behavior. Previous studies that used financial literacy and financial education as proxies for rational decisions to show that they are associated with positive health behavior, support our hypothesis. To the best of our knowledge, no previous study has addressed the relationship between financial literacy, financial education, and cancer screening. Most of the articles focus on the cost-effectiveness aspect of cancer screening programs [27,28,29], while others focus on accessibility disparities between people with different levels of education and income [14,30,31,32], or exploring possible links between factors such as psychological distress [33], cancer stigma [15], and cancer screening perceptions. To fill this gap, we investigate cancer screening behavior from the perspective of rational decision-making framework using financial literacy and financial education as proxies for rationality and contribute to the literature in at least two ways. Firstly, not only is this the first study to examine the relationship between financial literacy, financial education, and cancer screening activity among the Japanese population, but our study also provides incremental evidence on the role of financial literacy and financial education as rational decision-making tools in influencing people’s cancer screening behavior. Secondly, our study recommends the development of sustainable and effective cancer screening policies that can ultimately improve cancer screening participation in Japan.

## 2. Data and Methods

### 2.1. Data

Our study used data from the 2010 and 2011 waves of the Preference Parameters Study (PPS) of the Institute of Social and Economic Research at Osaka University, which was carried out annually over a period of 10 years, collecting prospective information of Japanese individuals on socioeconomic characteristics and preferences. We used the 2010 dataset to extract information on financial literacy and financial education as the main explanatory variables, while the 2011 wave was included to obtain data related to cancer screening behavior as a dependent variable and data on other control variables. Only the PPS survey in 2010 had questions on financial literacy and financial education.

According to the most updated guidelines in Japan [1], those above 40 years of age are the main target participants; therefore, our sample only includes Japanese individuals who are 40 years old or older. The 2 datasets were merged using panel identification information from each respondent, involving 3403 people after the unmatched data were removed. We excluded 710 individuals under 40; thus, our final sample comprised 2693 responses, accounting for 54.58% of the total responses in the 2011 dataset (4943 observations).

### 2.2. Variables

The dependent variable was the number of cancer screenings; 1 question in the 2011 PPS dataset enquires about cancer screening behavior—“[Over the last 12 months] Have you had any cancer examinations?”—with 6 possible answers including stomach, lung, uterine, breast, colon, and other cancer examinations as well as a seventh response option of not having taken any cancer examination. We created the dependent variable of the number of cancer screenings—an ordinal variable that summed data from all types of cancer, other than breast and uterine cancers, which were excluded to prevent any effect of these gender-specific cancer types on the associations between variables. However, our data do not specify the type of cancer in “other cancer examinations”, which could include gender-specific cancers such as prostate cancer. We believe that this possible inclusion would not significantly affect the results of our study as only 6.05% of the respondents had chosen “other cancer examinations”, of which 4.7% were men.

Our study consisted of two principal explanatory variables: financial literacy and financial education. The 2010 PPS dataset contains 3 questions to measure financial literacy following the methodology of Lusardi and Mitchell [34]. These questions measure respondents’ ability to understand the implications of interest rates, inflation, and risk diversification. Similar to studies that used the same questions [9,10,35,36,37,38,39], we adopted 1 as the score for every correct answer and 0 for an incorrect answer and averaged scores of the total 3 responses to obtain data measuring financial literacy levels.

In terms of financial education, relevant data were extracted from the 2010 PPS question: “Did you receive any compulsory financial education when you were in high school?” We assigned a score of 1 to “yes” and 0 to “no” and “do not know.” In our study, one noticeable difference between the terms financial literacy and financial education study was that while the former tested financial knowledge from an investment point of view, the latter concentrated on savings behavior. Specifically, financial education has become a part of the Japanese primary education curriculum, delivering lessons on savings by organizing a bank campaign targeted at children [40,41]. Financial education has been shown to exert no impact on financial literacy among Japanese populations who had already received this type of teaching [42], rendering it reasonable to evaluate both terms.

Other demographic and socioeconomic variables were gender, age, university degree, marital status, number of household members, having any children, employment status, household income, and household assets. Risky health behaviors included smoking, alcohol consumption, and frequent gambling. Other control variables were a myopic view of the future, happiness level, health anxiety, and poor health status. The definitions of all variables are summarized in Table 1.

### 2.3. Descriptive Statistics

Table 2 shows the description of main variables. The number of cancer screening tests undertaken by an average person was 1.1508 (range: 0–4). Average financial literacy was 0.62, while 17.56% of the sample received compulsory financial education during primary school years. Around 49.98% of the respondents were males, the average age was 55 (range: 40–77), and 25% respondents attained university education or higher. Nearly 87% were married, compared to only a minority of 4% divorced; each household had an average of 3 persons and 88% of the respondents had at least one child. The proportion of unemployed respondents was only 2%. Annual household income was an average of 6,523,023 Japanese yen, while the figure for household assets was 14,500,000 Japanese yen. Regarding risky health behavior, just under 22% of the subjects occasionally smoked or consumed more than one pack of cigarettes, while almost 48% drank alcohol. The percentage of people who gambled once a week or more frequently was almost 10%. While the majority of respondents (63.8%) felt happy with their current lives, 16.8% had a myopic view of the future. Although 43% felt anxious and had a great deal of concern about their health, only 2% self-described their health conditions as poor.

Table 3, Table 4, Table 5 and Table 6 show remarkable differences between cancer screening behavior based on various characteristics. Table 3 highlights the discrepancy of cancer screening participation among three main age categories. Overall, just under half of the surveyed people did not undergo cancer screening in 2011, while the proportion of cancer screening participants increased steadily with age. Thus, age-related factors can exert a substantial impact on cancer screening behavior. The highest percentage of screened respondents were in the 50–59 group with 3 tests, while the lowest were in the age cohort of 40–49 with 4 tests.

Table 4 shows marked differences between cancer screening behavior based on the three main demographic characteristics. The percentage of male respondents taking cancer screening tests was substantially higher than that of their female counterparts, and the highest figures for both genders were seen in those taking 3 cancer tests, at 18.34% for women and 20.73% for men. Likewise, a dramatic gap was observed between people at different educational levels, with higher education levels corresponding to more screening tests taken, except for those getting four tests. Of those who did not undergo cancer screening, more than half (51.43%) obtained lower than university qualifications. A similar and significant difference was observed in the unemployment variable.

**Table 4:** Distribution of the number of cancer screenings by demographic characteristics.

Number of Cancer Screenings	Gender		Education		Unemployed		Total
	Female	Male	Below University Degree	Above University Degree	No	Yes	
0	723	588	1043	268	1276	35	1311
	53.67%	43.68%	51.43%	40.3%	48.37%	63.64%	48.68%
1	173	192	267	98	357	8	365
	12.84%	14.26%	13.17%	14.74%	13.53%	14.55%	13.55%
2	187	217	285	119	397	7	404
	13.88%	16.12%	14.05%	17.89%	15.05%	12.73%	15%
3	247	279	372	154	523	3	526
	18.34%	20.73%	18.34%	23.16%	19.83%	5.45%	19.53%
4	17	70	61	26	85	2	87
	1.26%	5.2%	3.01%	3.91%	3.22%	3.64%	3.23%
Total	1347	1346	2028	665	2638	55	2693
	100%	100%	100%	100%	100%	100%	100%
Mean difference	t = 5.7938 ***		t = −4.7235 ***		t = 2.5508 **		

Note: *** *p* < 0.01, ** *p* < 0.05.

Dramatic differences between cancer screening behavior and the two major risky health habits, smoking and alcohol consumption, are illustrated in Table 5. Specifically, the proportion of screened smokers was lower than that of screened nonsmokers at the 1% significance level, regardless of the number of screenings. On the contrary, a higher probability of cancer screening among drinkers was observed in those taking more than two cancer tests. However, we did not find a sizeable disparity from the perspective of frequent gamblers.

**Table 5:** Distribution of the number of cancer screenings by risky health behavior.

Number of Cancer Screenings	Current Smoker		Current Drinker		Frequent Gambler		Total
	No	Yes	No	Yes	No	Yes	
0	981	330	716	595	1180	131	1311
	46.49%	56.6%	50.78%	46.38%	48.58%	49.62%	48.68%
1	289	76	204	161	324	41	365
	13.7%	13.04%	14.47%	12.55%	13.34%	15.53%	13.55%
2	327	77	204	200	361	43	404
	15.5%	13.21%	14.47%	15.59%	14.86%	16.29%	15%
3	444	82	253	273	488	38	526
	21.04%	14.07%	17.94%	21.28%	20.09%	14.39%	19.53%
4	69	18	33	54	76	11	87
	3.27%	3.09%	2.34%	4.21%	3.13%	4.17%	3.23%
Total	2110	583	1410	1283	2429	264	2693
	100%	100%	100%	100%	100%	100%	100%
Mean difference	t = 4.4427 ***		t = −3.5599 **		t = 0.9381		

Note: *** *p* < 0.01, ** *p* < 0.05.

Cancer screening behavior varied among people with different financial literacy scores (Table 6). The proportion of those with higher financial literacy (scores greater than 0.5) accessing cancer screening was considerably greater than that of the remaining group at the 1% significance level. Similarly, 22.2% of people who received financial education in primary schools participated in 3 cancer screening tests, which was much higher than the figure for those who were not financially educated.

**Table 6:** Distribution of the number of cancer screenings by financial literacy and financial education levels.

Number of Cancer Screenings	Financial Literacy		Financial Education		Total
	Score < 0.5	Score ≥ 0.5	No	Yes	
0	470	841	1104	207	1311
	52.93%	46.59%	49.73%	43.76%	48.68%
1	110	255	305	60	365
	12.39%	14.13%	13.74%	12.68%	13.55%
2	132	272	325	79	404
	14.86%	15.07%	14.64%	16.7%	15%
3	159	367	421	105	526
	17.91%	20.33%	18.96%	22.2%	19.53%
4	17	70	65	22	87
	1.91%	3.88%	2.93%	4.65%	3.23%
Total	888	1805	2220	473	2693
	100%	100%	100%	100%	100%
Mean difference	t = −3.2526 ***		t = −2.9948 **		

Note: *** *p* < 0.01, ** *p* < 0.05.

### 2.4. Methodology

The effects of financial literacy and financial education on cancer screening behavior, using the ordered probit regression method, are estimated separately in Equations (1) and (2), respectively. This was to accurately ascertain the sign, significance, and magnitude at which each of these variables was related to preventive cancer screening. Finally, financial literacy and financial education were included in the model to examine the combined effects of the variables in Equation (3).

(1) $$Y_{i}=f(FL_{i},\;X_{i},\;\varepsilon_{i})$$

(2) $$Y_{i}=f(FE_{i},\;X_{i},\;\varepsilon_{i}),$$

(3) $$Y_{i}=f(FL_{i},\;FE_{i},\;X_{i},\;\varepsilon_{i}),$$

where $Y_{i}$ is the preventive cancer screening behavior of the ith respondent, FL represents the score on the financial questions measuring financial literacy, FE is the financial education status received at school, *X* is a vector of individual respondents’ characteristics, and ε is the error term.

A study by Watanapongvanich et al. [9] suggests that respondents with a higher education status are more likely to have better financial knowledge, higher income, and more assets. Thus, independent variables such as financial literacy, university degree, household income, and household assets might be correlated. Therefore, a variance inflation test (VIF test) was performed to examine the correlation between the variables, the results of which are available upon request. Our VIF value was below 10; therefore, our study did not have multicollinearity issues.

To examine the determinants of cancer screening behavior, the following full specifications were considered, which were derived from Equations (1)–(3), respectively:

(4) $$Number\;of\;cancer\;screenings_{i}=\beta_{0}+\beta_{1}financial\;literacy_{i}+\beta_{2}male_{i}+\;\beta_{3}age_{i}+\beta_{4}university\;degree_{i}+\beta_{5}marriage_{i}+\beta_{6}divorce_{i}+\;\beta_{7}household\;size_{i}+\beta_{8}children_{i}+\beta_{9}unemployed_{i}+\;\beta_{10}\log ofhousehold\;income_{i}+\beta_{11}\log of\;household\;assets_{i}+\;\beta_{12}current\;smoker_{i}+\beta_{13}current\;drinker_{i}+\beta_{14}frequent\;gamblers_{i}+\;\beta_{15}myopic\;view\;of\;the\;future_{i}+\;\beta_{16}current\;level\;of\;the\;happiness_{i}+\;\beta_{17}anxiety\;about\;health_{i}+\beta_{18\;}poor\;health\;status_{i}+\;\varepsilon_{i}$$

(5) $$Number\;of\;cancer\;screenings_{i}=\beta_{0}+\beta_{1}financial\;education_{i}+\beta_{2}male_{i}\;+\beta_{3}age_{i}+\beta_{4}university\;degree_{i}+\beta_{5}marriage_{i}+\beta_{6}divorce_{i}+\;\beta_{7}household\;size_{i}+\beta_{8}children_{i}+\beta_{9}unemployed_{i}+\;\beta_{10}\log ofhousehold\;income_{i}+\beta_{11}\log of\;household\;assets_{i}+\;\beta_{12}current\;smoker_{i}+\beta_{13}current\;drinker_{i}+\beta_{14}frequent\;gamblers_{i}+\;\beta_{15}myopic\;view\;of\;the\;future_{i}+\beta_{16}current\;level\;of\;the\;happiness_{i}+\;\beta_{17}anxiety\;about\;health_{i}+\beta_{18\;}poor\;health\;status_{i}+\;\varepsilon_{i}$$

(6) $$Number\;of\;cancer\;screenings_{i}=\beta_{0}+\beta_{1}financial\;literacy_{i}+\;\beta_{2}financial\;education_{i}+\beta_{3}male_{i}+\beta_{4}age_{i}+\beta_{5}university\;degree_{i}+\;\beta_{6}marriage_{i}+\beta_{7}divorce_{i}+\beta_{8}household\;size_{i}+\beta_{9}children_{i}+\;\beta_{10}unemployed_{i}+\beta_{11}\log ofhousehold\;income_{i}+\;\beta_{12}\log of\;household\;assets_{i}+\beta_{13}current\;smoker_{i}+\beta_{14}current\;drinker_{i}\;+\beta_{15}frequent\;gamblers_{i}+\beta_{16}myopic\;view\;of\;the\;future_{i}+\;\beta_{17}current\;level\;of\;the\;happiness_{i}+\beta_{18}anxiety\;about\;health_{i}+\;\beta_{19\;}poor\;health\;status_{i}+\;\varepsilon_{i}$$

where $Number\;of\;cancer\;screenings_{i}$ is the dependent variable, illustrating the number of cancer screening tests undertaken. It is an ordinal variable within the range of 0 to 4, and its value was assigned by combining a simple count of the number of different cancer-type screening tests (breast and uterine cancers excluded). Therefore, we ran ordered probit models for Number of cancer screenings.

## 3. Results

The results of regression models showing the association of the number of cancer screenings with financial literacy, financial education, and both are reported in Table 7 and Table 8. Model 1 provides estimates for the relationship between financial literacy, Model 2 for financial education, and Model 3 for both. Model 1.1 comprises the main explanatory variable(s) and demographic characteristics. Model 1.2 includes the variables in Model 1.1 and other risky health behavior variables. Model 1.3 includes Model 1.2 with an additional myopic view of the future. Model 1.4 supplements three more variables to Model 1.3, namely “current level of happiness,” “anxiety about health,” and “poor health status.” The same controlled variables are included in Models 2.1–2.4 and Models 3.1–3.4.

Table 7 shows that financial literacy had a positive but insignificant impact on the number of cancer screenings across all models. Financial education had a positive and significant impact on the number of cancer screenings in all models at the 10% significance level. Table 8, which included both explanatory variables, shows no differences overall in the significance of the estimated parameters compared to the results in Table 7. Thus, respondents with a high level of financial education were more likely to participate in multiple cancer screenings, but we found no such relationship in the case of financial literacy. The signs and significance levels of all control variables were relatively consistent among all models and specifications (Table 7 and Table 8). We found that being male, older, having at least one child, higher levels of household income and household assets, and higher subjective feelings of happiness had a positive and significant relationship with the number of cancer screenings, while smoking was negatively related.

Table 9 shows the association of the number of cancer screenings with both financial literacy and financial education, according to 3 age groups (40–49, 50–59, and >60). Model 3.4.1 describes the results of the youngest age group (40–49), followed by Model 3.4.2 for those aged 50–59 and Model 3.4.3 for the oldest age group (60 plus).

As shown in Table 9, the effects of other control variables were not robust and consistent among the three age cohorts. Financial education had a statistically significant impact on the number of cancer screenings only in the 50–59 age group. Financial literacy did not have any effect on the behavior of interest, regardless of age category.

## 4. Discussion

We provide evidence that financial education is positively associated with cancer screening behavior, implying that financially educated people are more likely to participate in cancer screening. This is consistent with Grossman’s human capital model [11]. Specifically, engaging in cancer screening exhibits demand for medical care, a heavy investment in health capital, and a catalyst for productivity enhancement and utility maximization. While the role of financial education in savings, investment, and economic growth is already evident [43], we provide incremental evidence that financially educated people would respond rationally to cancer screening, as this action would take them closer to meeting basic demand for health care and achieving good health. However, we did not find any significant association between financial literacy and preventive cancer screening. This is probably because financial literacy, as measured in our study, focused more on investment aspects, while financial education emphasized saving behavior. Many studies have confirmed financial education as a proxy for rationality from the perspective of enhanced saving behavior [42,44,45], which means that financially educated people are less likely to procrastinate their savings plans [46]. Since procrastination is one of the reasons for the lack of screening [47,48], it is justified that financially educated people are more likely to participate in cancer screening. This provides clear evidence on the effect of financial education as a constructive tool to strengthen rational decision-making ability to avoid delaying cancer screening tests. Furthermore, Lentz et al. [49] reinforce the view that cancer and its accompanying treatment can result in the depletion of savings, which would also be improved with financial education programs [45]. Our study also showed that the association between financial education and cancer screening was the strongest in the 50–59 age cohort. This phenomenon could be explained by the fact that the influence of financial education is largely observed when it is provided at a teachable moment [50] and that the influence of financial education on specific behavior decays over time [51]. We would argue that financial education for those at targeted age groups of cancer screening, especially 50- to 59-year-olds, plays a key role in improving preventive cancer screening in Japan.

In terms of demographic variables, our study found that men have a higher tendency to participate in cancer screening and take more cancer screening tests than women. The results of our study are similar to those of Davis et al. [52], who concluded that men show a greater willingness toward cancer screening under the condition of being elaborately consulted about the tests. Other studies also found that men are more likely to attend cancer screening than women because of their higher privileges of socioeconomic backgrounds and lower barriers to screening facilities [53,54]. In fact, in the Japanese society, men play a dominant role in the workforce, compared to the opposite sex, which provides Japanese males with the opportunity to easily access cancer screening services during mandatory health-checkup periods at the workplace. In this case, the discrepancy in the tendency to reach cancer screening between two genders can be attributed to healthcare accessibility, rather than health consciousness. However, a meta-analysis revealed a lower uptake of health screening in men for prostate cancer [55], which is a compelling reason for the mixed evidence on men’s adherence to cancer screening. Our study showed that the older people become, the greater the chance they will attend cancer screening tests. This is understandable because older people run a higher risk of contracting noncommunicable diseases, especially cancer, which implies the necessity of screening for cancer. This result is consistent with findings from Kressin et al. [56] and Starker and Saß [57], which indicate a higher willingness to undergo cancer screening and higher participation rates along with increasing age, respectively. Marriage and divorce were not associated with cancer screening behavior. Although this insignificant association does not align with other studies that reveal a higher tendency of cancer screening participation among married people [58,59], the difference may be due to measurement differences; thus, a future prospective study to test this association is warranted. Regarding the variables “household size” and “child,” while no significant and consistent results were recorded, we found a positive link between having children and cancer screening only among 50- to 59-year-olds. This is in contrast to the results of another study [60], which was only applicable to families with children under 18. In fact, with the introduction of the 21 National Campaign for Healthy Parents since 2000 [61], Japanese parents may be more willing to spend time and money on their health, particularly for screening for cancer.

Socioeconomic variables included in our study were educational level, unemployment, household income, and household assets. Generally, previous studies have shown that individuals with a high SES are more inclined to be screened than those with a low SES [62,63]. For educational levels, we found that university qualification only had a significantly positive relationship with cancer screening among 40- to 49-year-olds, while a negative association was even seen in those over 60 years of age. This inconsistency is supported by other studies by Kim et al. [64] and Son [65], who found no association between educational levels and cancer screening in Korea. This is probably because opportunities for screening services are widely available in different health settings in Japan, and each resident can easily take these tests under the coverage of either national health insurance or employment-based insurance systems. Consistent with [62], a significant negative relationship between unemployment and cancer screening behavior was seen, particularly among 40- to 49-year-olds. In Japan, the issue of cancer screening is closely related to work [66], where screening services are within the reach of workers in medium and large corporations, while accessibility is less for those working in small companies and jobless people. Combined with dramatically higher unemployment rates in the age group 40–49 compared to those aged 50 years and older [67], joblessness in this age range undoubtedly has a much larger effect on the relationship with cancer screening behavior. For household income and total assets, we find robust and consistent positive significance between these variables and cancer screening in the three main age groups. This is in agreement with other studies [58,59,62,68], which showed higher rates of cancer screening among households with higher incomes.

Smoking and alcohol consumption were the two risky health behaviors included in this study. While alcohol consumption was not related to cancer screening, smoking was negatively related. This is consistent with various findings by Kim et al. [69], Kim et al. [64], and Martires et al. [70] that smokers are less likely to participate in screening programs, perhaps because they are not willing to ask for a consultation for health prevention [70] or they are already aware of many adverse health events when smoking and are afraid of receiving negative results [71]. Meanwhile, no significant differences were observed in terms of alcohol consumption reported in another study [69].

Finally, we found no significant effect of frequent gambling, a myopic view of the future, anxiety about health, or poor health status on cancer screening behavior. These findings are consistent with those of previous studies on the inconsistent and insignificant association between cancer screening and health anxiety [72,73] and poor health status [64,74,75]. We reported a positive association between the feeling of happiness and cancer screening behavior, particularly significant among the age group of 40–49-year-olds. This is supported by Goel et al. [76], who claimed that those with less satisfaction with life may be more common among those with lower socioeconomic status, thus being more associated with unwillingness to check for health screening considering the high cost of healthcare utilization later on [77,78].

This study had several limitations. Firstly, although we utilized the PPS dataset, which is a panel survey from 2003 to 2013, we could only analyze the 2011 wave, as it provided information on the number of cancer screening tests taken within a year. This study does not provide longitudinal evidence on the association between financial education and preventive cancer screening, which means that the causality of the partial relationship reported from our results has room for improvement. Therefore, future prospective studies should be conducted to establish this relationship. Secondly, our study does not consider breast and uterine cancer screening, as these are gender-specific screening programs, and this factor may have become a confounding factor in the overall association. Thus, the evaluation of the relationship between financial literacy, financial education, and cancer screening behavior among women is needed in the future. Third, our measurement of financial literacy was restricted to three questions, while financial education was assessed using only one question. While alternative measurements of financial literacy and financial education are available, a large body of related studies adopted the same methodology as that used in our study [8,9,10].

## 5. Conclusions

Despite several initiatives, cancer screening rates are still low in Japan and have scope for improvement. We observed cancer screening behavior from the viewpoint of a rational decision-making framework and hypothesized that financial literacy and financial education are positively related to cancer screening. While cancer screening was positively associated with financial education, we found no association between financial literacy and cancer screening. Our results imply that financial education is likely to enable people to behave rationally and help them to understand the value of preventive cancer screening, which ultimately serves as an investment in health capital. The results have important implications for raising awareness of Japanese populations to have cancers screened. In addition to conventional approaches to encourage people to participate in cancer screening, such as sending vouchers or organizing health educational programs related to the benefits of health screening, it is worth considering intervening the issue from the perspective of rationality. When people acquire the ability to make rational behavioral decisions, there is a greater chance of recognizing the value of cancer screening as a sustainable investment in health capital. Thus, policymakers could consider promoting financial education campaigns nationwide as an alternative measure to enhance cancer screening participation. Moreover, a financial education program could be implemented at the school level to motivate students to make rational health decisions such as preventive cancer screening at the later stage of their life. Future studies in this field should be directed toward providing longitudinal evidence on the relationship between financial education and preventive cancer screening behavior to provide more concrete evidence and policy implications.

## Figures and Tables

**Table 1:** Variable definitions.

Variables	Definitions
Number of cancer screenings	Ordinal variable: number of different cancer types screening tests (except breast, uterine cancer) taken within 1 year
Financial literacy	Continuous variable: average score for number of correct answers from the following three financial literacy questions: 1. Suppose you had 10,000 JPY in a savings account and the interest rate is 2% per year and you never withdraw money or interest payments. After 5 years, how much would you have in this account in total? 2. Imagine that the interest rate on your savings account is 1% per year and inflation is 2% per year. After 1 year, how much would you be able to buy with the money in this account? 3. Please indicate whether the following statement is true or false. “Buying a company stock usually provides a safer return than a stock mutual fund”.
Financial education	Binary variable: 1 = received compulsory financial education at school, 0 = otherwise
Male	Binary variable: 1 = male, 0 = female
Age	Age of participants
University degree	Binary variable: 1 = obtained a university degree or higher, 0 = otherwise
Marriage	Binary variable: 1 = married, 0 = otherwise
Divorce	Binary variable: 1 = divorced, 0 = otherwise
Household size	The number of people living in the household
Children	Binary variable: 1 = have at least one child, 0 = otherwise
Unemployed	Binary variable: 1 = unemployed, 0 = otherwise
Household income	Annual earned income before taxes and with bonuses of entire household in 2010 (JPY)
Log of household income	Log (household income)
Household assets	The balanced amount of financial assets (savings, stocks, insurance, etc.) of entire household (JPY)
Log of household assets	Log (household assets)
Current smoker	Binary variable: 1 = smoke (occasionally–more than one pack a day), 0 = do not smoke (never smoke, hardly smoke, already quit smoking)
Current drinker	Binary variable: 1 = drink (sometimes-5 cans a day), 0 = do not drink (do not drink at all, hardly drink)
Frequent gambler	Binary variable: 1 = frequent gambler (once a week or more), 0 = otherwise
Myopic view of the future	Binary variable: 1 = agree/completely agree with “Since the future is uncertain, it is a waste to think about it”, 0 = otherwise
Current level of happiness	Continuous variable: percentage score from the question “Overall, how happy would you say you are currently?”
Anxiety about health	Binary variable: 1 = agree/completely agree with “I have anxiety about my health”, 0 = otherwise
Poor health status	Binary variable: 1 = describe current health status as poor, 0 = otherwise

**Table 2:** Descriptive statistics.

Variable	Mean	Standard Deviation (SD)	Min	Max
Main variables				
Number of cancer screenings	1.1508	1.2988	0	4
Financial literacy	0.6206	0.3341	0	1
Financial education	0.1756	0.3806	0	1
Other variables				
Male	0.4998	0.5001	0	1
Age	55.1381	9.2772	40	77
University degree	0.25	0.43	0	1
Marriage	0.8652	0.3416	0	1
Divorce	0.0423	0.2014	0	1
Household size	3.43	1.43	1	10
Children	0.8819	0.3228	0	1
Unemployed	0.0204	0.1415	0	1
Household income	6,523,023	3,845,144	1,000,000	20,000,000
Log of household income	15.5161	0.6151	13.8155	16.8112
Household asset	14,500,000	18,100,000	2,500,000	100,000,000
Log of household asset	15.9348	1.0226	14.7318	18.4207
Current smoker	0.2165	0.4119	0	1
Current Drinker	0.4764	0.4995	0	1
Frequent gambler	0.0980	0.2974	0	1
Myopic view of the future	0.1682	0.3741	0	1
Current level of happiness	0.6384	0.1780	0	1
Anxiety about health	0.4300	0.4952	0	1
Poor health status	0.0167	0.1282	0	1
Observations	2693			

**Table 3:** Distribution of the number of cancer screenings by age group.

Number of Cancer Screenings	Age			Total
	40–49	50–59	≥60	
0	495	400	416	1311
	55.93%	47.45%	43.11%	48.68%
1	114	113	138	365
	12.88%	13.4%	14.3%	13.55%
2	113	123	168	404
	12.77%	14.59%	17.41%	15%
3	152	182	192	526
	17.18%	21.59%	19.9%	19.53%
4	11	25	51	87
	1.24%	2.97%	5.28%	3.23%
Total	885	843	965	2693
	100%	100%	100%	100%
Mean difference	F = 17.63 ***			

Note: *** *p* < 0.01.

**Table 7:** Ordered probit model regression results with either financial literacy or financial education as the main explanatory variable.

Variables	Dependent Variable: Number of Cancer Screenings							
	Financial Literacy as Main Explanatory Variable				Financial Education as Main Explanatory Variable			
	Model 1.1	Model 1.2	Model 1.3	Model 1.4	Model 2.1	Model 2.2	Model 2.3	Model 2.4
Financial literacy	0.056	0.0522	0.0486	0.04				
	(−0.0676)	(−0.0676)	(−0.0675)	(−0.0677)				
Financial education					0.103 *	0.104 *	0.104 *	0.110 *
					(−0.0571)	(−0.0569)	(−0.0569)	(−0.0572)
Male	0.224 ***	0.286 ***	0.284 ***	0.291 ***	0.232 ***	0.293 ***	0.291 ***	0.298 ***
	(−0.0467)	(−0.0514)	(−0.0514)	(−0.0517)	(−0.0462)	(−0.0511)	(−0.0511)	(−0.0514)
Age	0.0152 ***	0.0142 ***	0.0144 ***	0.0144 ***	0.0147 ***	0.0137 ***	0.0139 ***	0.0140 ***
	(−0.00279)	(−0.0028)	(−0.0028)	(−0.00282)	(−0.00279)	(−0.0028)	(−0.0028)	(−0.00282)
University degree	0.0985 *	0.071	0.0691	0.0672	0.103 *	0.0747	0.0724	0.0694
	(−0.0535)	(−0.0539)	(−0.0539)	(−0.0541)	(−0.0529)	(−0.0533)	(−0.0532)	(−0.0534)
Marriage	0.141	0.124	0.125	0.0913	0.137	0.12	0.121	0.0859
	(−0.0945)	(−0.0945)	(−0.0944)	(−0.0954)	(−0.0944)	(−0.0945)	(−0.0943)	(−0.0953)
Divorce	0.0904	0.114	0.119	0.0882	0.098	0.121	0.126	0.095
	(−0.141)	(−0.141)	(−0.141)	(−0.142)	(−0.141)	(−0.141)	(−0.141)	(−0.142)
Household size	−0.00952	−0.00993	−0.0104	−0.00747	−0.011	−0.0114	−0.0117	−0.00859
	(−0.0181)	(−0.0181)	(−0.0182)	(−0.0182)	(−0.018)	(−0.0181)	(−0.0181)	(−0.0182)
Children	0.217 ***	0.211 ***	0.212 ***	0.206 **	0.214 ***	0.208 **	0.208 ***	0.201 **
	(−0.0802)	(−0.0808)	(−0.0807)	(−0.081)	(−0.0804)	(−0.0809)	(−0.0808)	(−0.0811)
Unemployed	−0.131	−0.133	−0.127	−0.103	−0.13	−0.132	−0.125	−0.1
	(−0.167)	(−0.169)	(−0.169)	(−0.169)	(−0.166)	(−0.169)	(−0.168)	(−0.168)
Log of household income	0.209 ***	0.207 ***	0.207 ***	0.188 ***	0.211 ***	0.209 ***	0.208 ***	0.188 ***
	(−0.0431)	(−0.0433)	(−0.0433)	(−0.044)	(−0.0431)	(−0.0433)	(−0.0433)	(−0.044)
Log of household assets	0.0732 ***	0.0645 ***	0.0631 ***	0.0543 **	0.0754 ***	0.0665 ***	0.0649 ***	0.0553 **
	(−0.0241)	(−0.0244)	(−0.0244)	(−0.0248)	(−0.0239)	(−0.0241)	(−0.0241)	(−0.0245)
Current smoker		−0.274 ***	−0.271 ***	−0.260 ***		−0.276 ***	−0.273 ***	−0.261 ***
		(−0.0574)	(−0.0574)	(−0.0574)		(−0.0575)	(−0.0574)	(−0.0575)
Current drinker		0.0539	0.0526	0.0475		0.0544	0.053	0.0475
		(−0.0473)	(−0.0474)	(−0.0474)		(−0.0473)	(−0.0474)	(−0.0475)
Frequent gambler		−0.0608	−0.0588	−0.059		−0.0544	−0.0525	−0.0521
		(−0.0743)	(−0.0743)	(−0.0743)		(−0.0744)	(−0.0744)	(−0.0744)
Myopic view of the future			−0.0732	−0.0724			−0.074	−0.0728
			(−0.0593)	(−0.0592)			(−0.0592)	(−0.0592)
Current level of happiness				0.380 ***				0.392 ***
				(−0.136)				(−0.136)
Anxiety about health				0.0521				0.0502
				(−0.0451)				(−0.0451)
Poor health status				−0.108				−0.122
				(−0.178)				(−0.178)
Constant cut1	5.660 ***	5.409 ***	5.369 ***	5.179 ***	5.684 ***	5.427 ***	5.383 ***	5.174 ***
	(−0.676)	(−0.679)	(−0.681)	(−0.687)	(−0.675)	(−0.677)	(−0.679)	(−0.685)
Constant cut2	6.020 ***	5.771 ***	5.731 ***	5.542 ***	6.044 ***	5.789 ***	5.746 ***	5.537 ***
	(−0.677)	(−0.68)	(−0.682)	(−0.687)	(−0.676)	(−0.678)	(−0.68)	(−0.685)
Constant cut3	6.469 ***	6.223 ***	6.185 ***	5.996 ***	6.494 ***	6.242 ***	6.199 ***	5.991 ***
	(−0.678)	(−0.681)	(−0.683)	(−0.688)	(−0.677)	(−0.679)	(−0.681)	(−0.686)
Constant cut4	7.617 ***	7.377 ***	7.338 ***	7.152 ***	7.643 ***	7.397 ***	7.354 ***	7.148 ***
	(−0.676)	(−0.679)	(−0.681)	(−0.686)	(−0.675)	(−0.677)	(−0.679)	(−0.684)
Observations	2693	2693	2693	2693	2693	2693	2693	2693
Log pseudolikelihood	−3510	−3497	−3496	−3492	−3508	−3496	−3495	−3490
Χ^2^ statistics	168.1	194.3	197.1	202.4	169.9	196.8	199.9	205.6
*p*-value	0	0	0	0	0	0	0	0

Robust standard errors in parentheses. *** *p* < 0.01, ** *p* < 0.05, * *p* < 0.1.

**Table 8:** Ordered probit model regression results with both financial literacy and financial education as the main explanatory variables.

Variables	Dependent Variable: The Number of Cancer Screening.			
	Model 3.1	Model 3.2	Model 3.3	Model 3.4
Financial literacy	0.0541	0.0503	0.0467	0.0398
	(−0.0675)	(−0.0675)	(−0.0674)	(−0.0676)
Financial education	0.102 *	0.104 *	0.103 *	0.110 *
	(−0.0571)	(−0.0569)	(−0.0569)	(−0.0572)
Male	0.227 ***	0.288 ***	0.286 ***	0.294 ***
	(−0.0467)	(−0.0515)	(−0.0514)	(−0.0517)
Age	0.0148 ***	0.0137 ***	0.0140 ***	0.0140 ***
	(−0.0028)	(−0.00281)	(−0.0028)	(−0.00282)
University degree	0.0970 *	0.0695	0.0676	0.0655
	(−0.0535)	(−0.0539)	(−0.0539)	(−0.054)
Marriage	0.137	0.12	0.121	0.0864
	(−0.0944)	(−0.0945)	(−0.0943)	(−0.0953)
Divorce	0.0964	0.119	0.125	0.0941
	(−0.141)	(−0.141)	(−0.141)	(−0.142)
Household size	−0.0101	−0.0105	−0.0109	−0.00793
	(−0.0181)	(−0.0181)	(−0.0181)	(−0.0182)
Children	0.213 ***	0.207 **	0.208 **	0.201 **
	(−0.0804)	(−0.0809)	(−0.0808)	(−0.0811)
Unemployed	−0.13	−0.132	−0.125	−0.101
	(−0.166)	(−0.168)	(−0.168)	(−0.168)
Log of household income	0.208 ***	0.207 ***	0.206 ***	0.187 ***
	(−0.0431)	(−0.0433)	(−0.0433)	(−0.044)
Log of household assets	0.0724 ***	0.0638 ***	0.0624 **	0.0533 **
	(−0.0242)	(−0.0244)	(−0.0244)	(−0.0248)
Current smoker		−0.276 ***	−0.273 ***	−0.261 ***
		(−0.0574)	(−0.0574)	(−0.0575)
Current drinker		0.0542	0.0529	0.0474
		(−0.0473)	(−0.0474)	(−0.0475)
Frequent gambler		−0.0545	−0.0525	−0.0522
		(−0.0744)	(−0.0744)	(−0.0744)
Myopic view of the future			−0.0723	−0.0714
			(−0.0592)	(−0.0592)
Current level of happiness				0.389 ***
				(−0.136)
Anxiety about health				0.0511
				(−0.0451)
Poor health status				−0.122
				(−0.178)
Constant cut1	5.626 ***	5.374 ***	5.335 ***	5.136 ***
	(−0.676)	(−0.679)	(−0.681)	(−0.686)
Constant cut2	5.986 ***	5.736 ***	5.698 ***	5.499 ***
	(−0.677)	(−0.679)	(−0.682)	(−0.687)
Constant cut3	6.436 ***	6.190 ***	6.151 ***	5.953 ***
	(−0.678)	(−0.68)	(−0.683)	(−0.688)
Constant cut4	7.586 ***	7.345 ***	7.306 ***	7.111 ***
	(−0.677)	(−0.678)	(−0.68)	(−0.685)
Observations	2693	2693	2693	2693
Log pseudolikelihood	−3508	−3495	−3495	−3490
Χ^2^ statistics	170.5	197.2	200.1	205.8
*p*-value	0	0	0	0

Robust standard errors in parentheses. *** *p* < 0.01, ** *p* < 0.05, * *p* < 0.1.

**Table 9:** Ordered probit model regression results for the number of cancer screenings, stratified by three age groups.

Variables	Dependent Variable: Number of Cancer Screenings		
	Model 3.4.1 (40–49)	Model 3.4.2 (50–59)	Model 3.4.3 (≥60)
Financial literacy	0.0473	0.0391	0.0292
	(−0.125)	(−0.128)	(−0.106)
Financial education	−0.00433	0.210 **	0.0779
	(−0.129)	(−0.0924)	(−0.0899)
Male	0.126	0.407 ***	0.368 ***
	(−0.0913)	(−0.0955)	(−0.0888)
Age	0.0354 ***	−0.0036	0.0136
	(−0.0137)	(−0.0138)	(−0.00843)
University degree	0.268 ***	0.0776	−0.168 *
	(−0.0932)	(−0.0937)	(−0.0977)
Marriage	−0.0116	−0.128	0.202
	(−0.198)	(−0.205)	(−0.137)
Divorce	0.129	−0.263	0.271
	(−0.254)	(−0.309)	(−0.219)
Household size	0.0148	−0.0207	−0.033
	(−0.035)	(−0.0305)	(−0.0311)
Children	0.218	0.339 **	0.14
	(−0.176)	(−0.149)	(−0.127)
Unemployed	−0.755 **	−0.0544	0.259
	(−0.312)	(−0.318)	(−0.257)
Log of household income	0.259 ***	0.146 *	0.197 ***
	(−0.0954)	(−0.0761)	(−0.0699)
Log of household assets	0.0211	0.0626	0.0625
	(−0.0539)	(−0.0428)	(−0.0384)
Current smoker	−0.197 **	−0.342 ***	−0.225 **
	(−0.0955)	(−0.103)	(−0.102)
Current drinker	0.0235	0.0423	−0.00132
	(−0.0855)	(−0.0842)	(−0.0822)
Frequent gambler	−0.142	−0.0956	0.0868
	(−0.132)	(−0.118)	(−0.146)
Myopic view of the future	−0.184	0.0499	−0.0961
	(−0.115)	(−0.106)	(−0.0925)
Current level of happiness	0.611 ***	0.17	0.440 *
	(−0.229)	(−0.257)	(−0.234)
Anxiety about health	0.0919	−0.0269	0.0773
	(−0.0824)	(−0.0824)	(−0.0743)
Poor health status	−0.242	−0.0631	−0.0937
	(−0.542)	(−0.355)	(−0.224)
Constant cut1	6.848 ***	3.456 ***	5.432 ***
	(−1.467)	(−1.341)	(−1.236)
Constant cut2	7.213 ***	3.813 ***	5.807 ***
	(−1.468)	(−1.342)	(−1.238)
Constant cut3	7.649 ***	4.248 ***	6.303 ***
	(−1.471)	(−1.342)	(−1.240)
Constant cut4	9.070 ***	5.494 ***	7.297 ***
	(−1.467)	(−1.342)	(−1.236)
Observations	885	843	965
Log pseudolikelihood	−1026	−1097	−1339
Chi^2^ statistics	100.2	61.11	68.03
*p*-value	0	2.58 × 10^−6^	1.95 × 10^−7^

Robust standard errors in parentheses. *** *p* < 0.01, ** *p* < 0.05, * *p* < 0.1.

## Data Availability

Data is available upon request.

## References

[1] Hamashima C. (2018). Cancer screening guidelines and policy making: 15 years of experience in cancer screening guideline development in Japan. Jpn. J. Clin. Oncol..

[2] Yoshida M., Kondo K., Tada T. (2010). The relation between the cancer screening rate and the cancer mortality rate in Japan. J. Med. Investig..

[3] Sano H., Goto R., Hamashima C. (2014). What is the most effective strategy for improving the cancer screening rate in Japan?. Asian Pac. J. Cancer Prev..

[4] Ueda Y., Sobue T., Morimoto A., Egawa-Takata T., Hashizume C., Kishida H., Okamoto S., Yoshino K., Fujita M., Enomoto T. (2015). Evaluation of a free-coupon program for cervical cancer screening among the young: A nationally funded program conducted by a local government in Japan. J. Epidemiol..

[5] Hamashima C., Sano H. (2018). Association between age factors and strategies for promoting participation in gastric and colorectal cancer screenings. BMC Cancer.

[6] Katanoda K., Ito Y., Sobue T. (2021). International comparison of trends in cancer mortality: Japan has fallen behind in screening-related cancers. Jpn. J. Clin. Oncol..

[7] Goto R., Hamashima C., Mun S., Lee W.C. (2015). Why screening rates vary between Korea and Japan—Differences between two national healthcare systems. Asian Pac. J. Cancer Prev..

[8] Ono S., Yuktadatta P., Taniguchi T., Iitsuka T., Noguchi M., Tanaka S., Ito H., Nakamura K., Yasuhara N., Miyawaki C. (2021). Financial literacy and exercise behavior: Evidence from Japan. Sustainability.

[9] Watanapongvanich S., Binnagan P., Putthinun P., Khan M.S.R., Kadoya Y. (2021). Financial literacy and gambling behavior: Evidence from Japan. J. Gambl. Stud..

[10] Yuktadatta P., Khan M.S.R., Kadoya Y. (2021). Financial literacy and exercise behavior in the United States. Sustainability.

[11] Grossman M. (1972). On the concept of health capital and the demand for health. J. Pol. Econ..

[12] Grossman M., Culyer A.J., Newhouse J.P. (2000). The Human Capital Model. Handbook of Health Economics.

[13] WHO-IARC (2021). Asia and cancers. The Global Cancer Observatory (GLOBOCAN).

[14] Shah S.A., Mahmood M.I., Ahmad N. (2020). Low socioeconomic status associated with poor cancer screening perceptions in Malaysia: Analysis of determinant of health among general population. Asian Pac. J. Cancer Prev..

[15] Vrinten C., Gallagher A., Waller J., Marlow L.A.V. (2019). Cancer stigma and cancer screening attendance: A population based survey in England. BMC Cancer.

[16] Cadet T.J., Burke S.L., Stewart K., Howard T., Schonberg M. (2017). Cultural and emotional determinants of cervical cancer screening among older Hispanic women. Health Care Women Int..

[17] Davis M., Oaten M., Occhipinti S., Chambers S.K., Stevenson R.J. (2017). An investigation of the emotion of disgust as an affective barrier to intention to screen for colorectal cancer. Eur. J. Cancer Care.

[18] Morgan P.J., Long T.Q. (2020). Financial literacy, financial inclusion, and savings behavior in Laos. J. Asian Econ..

[19] Yoshino N., Morgan P.J., Trinh L.Q. (2017). Financial Literacy in Japan: Determinants and Impacts.

[20] Bucher-Koenen T., Lusardi A. (2011). Financial literacy and retirement planning in Germany. J. Pension Econ. Fin..

[21] Lusardi A., Mitchell O.S. (2011). Financial literacy and retirement planning in the United States. J. Pension Econ. Fin..

[22] van Rooij M.C.J., Lusardi A., Alessie R.J.M. (2011). Financial literacy and retirement planning in the Netherlands. J. Econ. Psychol..

[23] van Rooij M.C.J., Lusardi A., Alessie R.J.M. (2012). Financial literacy, retirement planning and household wealth. Econ. J..

[24] Shankaran V., Linden H., Steelquist J., Watabayashi K., Kreizenbeck K., Leahy T., Overstreet K. (2017). Development of a financial literacy course for patients with newly diagnosed cancer. Am. J. Manag. Care.

[25] Benjamin D.J., Brown S.A., Shapiro J.M. (2013). Who is “behavioral”? cognitive ability and anomalous preferences. J. Eur. Econ. Assoc..

[26] James R. (2020). How Long Can You Have Cancer without Knowing about It?.

[27] Bretthauer M., Kalager M. (2013). Principles, effectiveness and caveats in screening for cancer. Br. J. Surg..

[28] Jun J.K., Choi K.S., Lee H.Y., Suh M., Park B., Song S.H., Jung K.W., Lee C.W., Choi I.J., Park E.C. (2017). Effectiveness of the Korean national cancer screening program in reducing gastric cancer mortality. Gastroenterology.

[29] Pavlik E.J. (2016). Ovarian cancer screening effectiveness: A realization from the UK collaborative trial of ovarian cancer screening. Womens Health.

[30] Rivera M.P., Katki H.A., Tanner N.T., Triplette M., Sakoda L.C., Wiener R.S., Cardarelli R., Carter-Harris L., Crothers K., Fathi J.T. (2020). Addressing disparities in lung cancer screening eligibility and healthcare access. An official American thoracic society statement. Am. J. Respir. Crit. Care Med..

[31] Warren Andersen S., Blot W.J., Lipworth L., Steinwandel M., Murff H.J., Zheng W. (2019). Association of race and socioeconomic status with colorectal cancer screening, colorectal cancer risk, and mortality in southern US adults. JAMA Netw. Open.

[32] Wools A., Dapper E.A., de Leeuw J.R. (2016). Colorectal cancer screening participation: A systematic review. Eur. J. Public Health.

[33] Fujiwara M., Inagaki M., Nakaya N., Fujimori M., Higuchi Y., Kakeda K., Uchitomi Y., Yamada N. (2018). Association between serious psychological distress and nonparticipation in cancer screening and the modifying effect of socioeconomic status: Analysis of anonymized data from a national cross-sectional survey in Japan. Cancer.

[34] Lusardi A., Mitchell O.S. (2008). Planning and financial literacy: How do women fare?. Am. Econ. Rev..

[35] Fornero E., Monticone C. (2011). Financial literacy and pension plan participation in Italy. J. Pension Econ. Fin..

[36] Kadoya Y., Khan M.S.R., Hamada T., Dominguez A. (2018). Financial literacy and anxiety about life in old age: Evidence from the USA. Rev. Econ. Household.

[37] Kadoya Y., Khan M.S.R. (2018). Can financial literacy reduce anxiety about life in old age?. J. Risk Res..

[38] Kadoya Y., Khan M.S.R. (2020). What determines financial literacy in Japan?. J. Pension Econ. Fin..

[39] Klapper L., Panos G.A. (2011). Financial literacy and retirement planning: The Russian case. J. Pension Econ. Fin..

[40] Messy F.-A., Monticone C. (2016). Financial Education Policies in Asia and the Pacific.

[41] Watanapongvanich S., Khan M.S.R., Putthinun P., Ono S., Kadoya Y. (2021). Financial literacy, financial education, and smoking behavior: Evidence from Japan. Front. Public Health.

[42] Sekita S. (2011). Financial literacy and retirement planning in Japan. J. Pension Econ. Fin..

[43] OECD (2006). The Importance of Financial Education.

[44] Amari M., Salhi B., Jarboui A. (2020). Evaluating the effects of sociodemographic characteristics and financial education on saving behavior. Int. J. Sociol. Soc. Policy.

[45] Lusardi A. (2007). Household Saving Behavior: The Role of Literacy, Information and Financial Education Programs.

[46] Brown J.R., Previtero A. Procrastination, present-biased preferences, and financial behaviors. Proceedings of the 16th Annual Joint Meeting of the Retirement Research Consortium.

[47] Green B.B., BlueSpruce J., Tuzzio L., Vernon S.W., Shay L.A., Catz S.L. (2017). Reasons for never and intermittent completion of colorectal cancer screening after receiving multiple rounds of mailed fecal tests. BMC Public Health.

[48] Lieberman A., Gneezy A., Berry E., Miller S., Koch M., Argenbright K.E., Gupta S. (2021). The effect of deadlines on cancer screening completion: A randomized controlled trial. Sci. Rep..

[49] Lentz R., Benson A.B., Kircher S. (2019). Financial toxicity in cancer care: Prevalence, causes, consequences, and reduction strategies. J. Surg. Oncol..

[50] Kaiser T., Menkhoff L. (2017). Does financial education impact financial literacy and financial behavior, and if so, when?. World Bank Econ. Rev..

[51] Fernandes D., Lynch J.G., Netemeyer R.G. (2014). Financial literacy, financial education, and downstream financial behaviors. Manag. Sci..

[52] Davis J.L., Buchanan K.L., Katz R.V., Green B.L. (2012). Gender differences in cancer screening beliefs, behaviors, and willingness to participate: Implications for health promotion. Am. J. Mens Health.

[53] Wardle J., Miles A., Atkin W. (2005). Gender differences in utilization of colorectal cancer screening. J. Med. Screen..

[54] Meissner H.I., Breen N., Klabunde C.N., Vernon S.W. (2006). Patterns of colorectal cancer screening uptake among men and women in the United States. Cancer Epidemiol. Biomark. Prev..

[55] Teo C.H., Ling C.J., Ng C.J. (2018). Improving Health Screening Uptake in Men: A Systematic Review and Meta-analysis. Am. J. Prev. Med..

[56] Kressin N.R., Manze M., Russell S.L., Katz R.V., Claudio C., Green B.L., Wang M.Q. (2010). Self-reported willingness to have cancer screening and the effects of sociodemographic factors. J. Natl. Med. Assoc..

[57] Starker A., Saß A.-C. (2013). Inanspruchnahme von Krebsfrüherkennungsuntersuchungen (Participation in Cancer Screening Programmes). Bundesgesundheitsbl.

[58] Fukuda Y., Nakamura K., Takano T. (2005). Reduced likelihood of cancer screening among women in urban areas and with low socio-economic status: A multilevel analysis in Japan. Public Health.

[59] Fukuda Y., Nakamura K., Takano T., Nakao H., Imai H. (2007). Socioeconomic status and cancer screening in Japanese males: Large inequality in middle-aged and urban residents. Environ. Health Prev. Med..

[60] Stimpson J.P., Wilson F.A., Reyes-Ortiz C.A. (2009). Influence of number of children on cancer screening among adults in the United States. J. Med. Screen..

[61] Osawa E., Ojima T., Akiyama Y., Yamagata Z. (2019). National campaign to promote maternal and child health in 21st century Japan. J. Natl. Inst. Public Health.

[62] Hahm M.I., Park E.C., Choi K.S., Lee H.Y., Park J.H., Park S. (2011). Inequalities in adoption of cancer screening from a diffusion of innovation perspective: Identification of late adopters. Cancer Epidemiol..

[63] Tabuchi T. (2020). Cancer and Socioeconomic Status. Springer Series on Epidemiology and Public Health.

[64] Kim Y.-H., Kim K., Han K.-d., Kim J.-S. (2015). Gender differences in elders’ participation in the national cancer screening program: Evidence from the korean national health and nutrition examination survey 2010–12. Iran. J. Public Health.

[65] Kwak M.-S., Park E.-C., Bang J.-Y., Sung N.-Y., Lee J.-Y., Choi K.-S. (2005). Factors associated with cancer screening participation, Korea. J. Prev. Med. Public Health.

[66] Brunner E., Cable N., Iso H. (2020). Health in Japan: Social Epidemiology of Japan Since the 1964 Tokyo Olympics.

[67] Kondo K. (2015). Spatial persistence of Japanese unemployment rates. Jpn. World Econ..

[68] Kim J.-Y., Kang H.-T. (2016). Association between socioeconomic status and cancer screening in Koreans over 40 years in age based on the 2010–2012 Korean national health and nutrition examination survey. Korean J. Fam. Med..

[69] Kim H.-J., Yim H.-W., Kim N.-C. (2014). Factors affecting cancer screening intention and behavior of the Korean elderly. Asian Pac. J. Cancer Prev..

[70] Martires K.J., Kurlander D.E., Minwell G.J., Dahms E.B., Bordeaux J.S. (2014). Patterns of cancer screening in primary care from 2005 to 2010. Cancer.

[71] Jonnalagadda S., Bergamo C., Lin J.J., Lurslurchachai L., Diefenbach M., Smith C., Nelson J.E., Wisnivesky J.P. (2012). Beliefs and attitudes about lung cancer screening among smokers. Lung Cancer.

[72] Jones S.M.W., Andersen M.R., Litwin P. (2022). Avoidance and reassurance seeking in response to health anxiety are differentially related to use of healthcare. J. Public Health.

[73] Knudsen A.K., Berge L.I., Skogen J.C., Veddegjærde K.-E., Wilhelmsen I. (2015). The prospective association between health anxiety and cancer detection: A cohort study linking the Hordaland Health Study (HUSK) with the norwegian cancer registry. J. Psychosom. Res..

[74] Lee S., Chen L., Jung M.Y., Baezconde-Garbanati L., Juon H.S. (2014). Acculturation and cancer screening among Asian Americans: Role of health insurance and having a regular physician. J. Community Health.

[75] Peeters N., Rijk K., Soetens B., Storms B., Hermans K. (2018). A Systematic literature review to identify successful elements for financial education and counseling in groups. J. Consum. Aff..

[76] Goel V., Rosella L.C., Fu L., Alberga A. (2018). The relationship between life satisfaction and healthcare utilization: A longitudinal study. Am. J. Prev. Med..

[77] Fitzpatrick T., Rosella L.C., Calzavara A., Petch J., Pinto A.D., Manson H., Goel V., Wodchis W.P. (2015). Looking beyond income and education: Socioeconomic status gradients among future high-cost users of health care. Am. J. Prev. Med..

[78] Rosella L.C., Fitzpatrick T., Wodchis W.P., Calzavara A., Manson H., Goel V. (2014). High-cost health care users in Ontario, Canada: Demographic, socio-economic, and health status characteristics. BMC Health Serv. Res..

